# Historical reconstruction of climatic and elevation preferences and the evolution of cloud forest-adapted tree ferns in Mesoamerica

**DOI:** 10.7717/peerj.2696

**Published:** 2016-11-16

**Authors:** Victoria Sosa, Juan Francisco Ornelas, Santiago Ramírez-Barahona, Etelvina Gándara

**Affiliations:** 1 Departamento de Biología Evolutiva, Instituto de Ecología AC, Carretera antigua a Coatepec, El Haya, Xalapa, Veracruz, Mexico; 2 Instituto de Ciencias/Herbario y Jardín Botánico, Benemérita Universidad Autónoma de Puebla, Puebla, Mexico

**Keywords:** Biogeography, Cloud forest, Tree ferns, Pleistocene, Cyatheaceae, Quaternary, Neogene, Elevational shifts, Climate tolerance, Neotropics

## Abstract

**Background:**

Cloud forests, characterized by a persistent, frequent or seasonal low-level cloud cover and fragmented distribution, are one of the most threatened habitats, especially in the Neotropics. Tree ferns are among the most conspicuous elements in these forests, and ferns are restricted to regions in which minimum temperatures rarely drop below freezing and rainfall is high and evenly distributed around the year. Current phylogeographic data suggest that some of the cloud forest-adapted species remained *in situ* or expanded to the lowlands during glacial cycles and contracted allopatrically during the interglacials. Although the observed genetic signals of population size changes of cloud forest-adapted species including tree ferns correspond to predicted changes by Pleistocene climate change dynamics, the observed patterns of intraspecific lineage divergence showed temporal incongruence.

**Methods:**

Here we combined phylogenetic analyses, ancestral area reconstruction, and divergence time estimates with climatic and altitudinal data (environmental space) for phenotypic traits of tree fern species to make inferences about evolutionary processes in deep time. We used phylogenetic Bayesian inference and geographic and altitudinal distribution of tree ferns to investigate ancestral area and elevation and environmental preferences of Mesoamerican tree ferns. The phylogeny was then used to estimate divergence times and ask whether the ancestral area and elevation and environmental shifts were linked to climatic events and historical climatic preferences.

**Results:**

Bayesian trees retrieved *Cyathea, Alsophyla, Gymnosphaera* and *Sphaeropteris* in monophyletic clades. Splits for species in these genera found in Mesoamerican cloud forests are recent, from the Neogene to the Quaternary, Australia was identified as the ancestral area for the clades of these genera, except for *Gymnosphaera* that was Mesoamerica. Climate tolerance was not divergent from hypothesized ancestors for the most significant variables or elevation. For elevational shifts, we found repeated change from low to high elevations.

**Conclusions:**

Our data suggest that representatives of Cyatheaceae main lineages migrated from Australia to Mesoamerican cloud forests in different times and have persisted in these environmentally unstable areas but extant species diverged recentrly from their ancestors.

## Introduction

A cloud forest is a moist tropical or subtropical montane type of vegetation characterized by a persistent, frequent or seasonal low-level cloud cover, usually at the canopy level (Foster, 2001; Holwerda et al., 2010; Ponette-González, Weathers & Curran, 2010a; Ponette-González, Weathers & Curran, 2010b). In eastern Mexico the cloud forest is restricted to ravines and patches along mountain slopes isolated by surrounding valleys and lowland plains (Rzedowski, 1996; Alcántara-Ayala, Luna-Vega & Velásquez, 2002). It is influenced by fog during the winter dry season, precipitation ranging from 1,000 to 3,000 mm and temperatures from 12 to 23°C (Holwerda et al., 2010; Ponette-González, Weathers & Curran, 2010a; Ponette-González, Weathers & Curran, 2010b; Rojas-Soto, Sosa & Ornelas, 2012). The area receives orographic precipitation usually produced by the prevailing winds from the northeast which sweep over this region dropping their moisture on windward slopes and edges of the mesas; drizzles and dense fogs during the drier months (November–March) create the conditions for certain species restricted to foggy areas (Miranda & Sharp, 1950). The seasonality of rain and fog enhance the development of exuberant vegetation, with a mixed canopy composed of temperate deciduous trees and tropical broadleaved-evergreen trees, a great variety of epiphytes and vines, and large tree ferns (Williams-Linera, 1997). Since the altitude band of cloud formation on tropical mountains is limited, cloud forest occurs in fragmented strips similar to island archipelagoes (Foster, 2001; Teale et al., 2014) and it is one of the most threatened habitats in the Neotropics. Currently covering <1% of the total area of vegetation in northern Mesoamerica, cloud forest is vulnerable to future climate change (Ponce-Reyes et al., 2012; Rehfeldt et al., 2012; Rojas-Soto, Sosa & Ornelas, 2012) and half of the original cloud forest area (pre-European) has been lost and replaced by other vegetation types (Luna-Vega et al., 2006; Muñoz Villers & López-Blanco, 2007).

In northern Mesoamerica, from southern Tamaulipas in Mexico to the Guatemalan highlands, cloud forest is the terrestrial ecosystem with highest diversity per unit area, high degree of epiphytes endemism, and includes 10% of Mexican flora (2,500 vascular plant species) and 12% of terrestrial vertebrates (Rzedowski, 1981; Rzedowski, 1996; Pineda & Halffter, 2004; Pineda & Halffter, 2004; Luna-Vega et al., 2006; Sánchez-González, Morrone & Navarro-Sigüenza, 2008). It is also characterized by a complex biogeographic history (Rzedowski, 1981), in which North American temperate-deciduous tree species that have been present in the region since the Tertiary are intermixed with tropical broadleaved-evergreen trees species with South American origins during the early Miocene (Miranda & Sharp, 1950; Sharp, 1951; Williams-Linera, 1997; Graham, 1999). The distributions of several North American temperate tree genera extend into Mexico and Central America, usually in cloud forests at higher elevations (Miranda & Sharp, 1950; Sharp, 1951; Williams-Linera, 1997; Graham, 1999; Qian & Ricklefs, 2004). In contrast, South American tropical tree genera extend into Mexico and Central America, usually in forests at lower altitudes (Williams-Linera, 1997). Several tree species in the region with temperate and tropical affinities are considered Tertiary relicts (Graham, 1999; Ornelas, Ruiz-Sanchez & Sosa, 2010; Ruiz-Sanchez & Ornelas, 2014). Paleoecological reconstruction in northern Mesoamerica indicates that the present disjunct distributions of temperate and montane cloud forest woody genera in Mexico and Central America have resulted from climate change during the late Tertiary and Pleistocene (Martin & Harrell, 1957; Graham, 1999), and that the colonization of tropical tree species and dynamics of cloud forests attributed solely to Pliocene processes has been obscured by Pleistocene climate changes (Figueroa-Rangel, Willis & Olvera-Vargas, 2010; Poelchau & Hamrick, 2013; Ramírez-Barahona & Eguiarte, 2014).

The dynamic Mesoamerican geological landscape from the Miocene to the Pliocene (Barrier et al., 1998; Manea et al., 2005) affected the distribution and composition of cloud forests in the region, and contributed to ancient divergences (e.g., Ornelas et al., 2013; Ruiz-Sanchez & Ornelas, 2014). Phylogeographical studies of Mesoamerican cloud forest-adapted species have emphasized temperate tree species that have migrated south from North America (reviewed in Jaramillo-Correa et al., 2009). The emerging phylogeographical patterns have been attributed to isolation by arid conditions during the Pliocene and climate changes during Pleistocene glaciations that promoted the expansion, contraction and divergence of populations (Jaramillo-Correa et al., 2009; Ruiz-Sanchez & Ornelas, 2014). Repeated cycles of cloud forest contraction and expansion due to Pleistocene climatic cycling shaped genetic divergence in the region, producing a common phylogeographical break at the Isthmus of Tehuantepec (Ornelas et al., 2013). However, the shared phylogeographical breaks of cloud forest-adapted taxa occurred as multiple vicariant events at different times (Ornelas et al., 2013). In contrast, plant species that presumably colonized cloud forest in the region from South America are little studied (Ornelas, Ruiz-Sanchez & Sosa, 2010; Gutiérrez-Rodríguez, Ornelas & Rodríguez-Gómez, 2011; Ramírez-Barahona & Eguiarte, 2014; Ornelas & Rodríguez-Gómez, 2015; Ornelas et al., 2016). Population expansion due to Pleistocene climatic cycling has been uncovered in some of these cloud forest-adapted species that presumably originated in South America (Gutiérrez-Rodríguez, Ornelas & Rodríguez-Gómez, 2011; Ramírez-Barahona & Eguiarte, 2014; Ornelas & Rodríguez-Gómez, 2015; Ornelas et al., 2016) but the times of interspecific and intraspecific lineage divergence are temporally incongruent (Ornelas et al., 2013; Ramírez-Barahona & Eguiarte, 2014).

Tree ferns (Cyatheales) are the second largest order of ferns (ca. 700 species; Smith et al., 2008) and one of the most conspicuous elements in cloud forests, where they grow in the sub-canopy with high tree cover (Pérez-Paredes, Sánchez-González & Tejero-Díaz, 2014). Eight families are considered within Cyatheales and all of them are distributed in the New World (Cibotiaceae, Culcitaceae, Cyatheaceae, Dicksoniaceae, Loxomataceae, Metaxyaceae, Plagiogyriaceae, and Thyrsopteridaceae; Smith et al., 2008). Among these scaly tree ferns (Cyatheaceae) are the most diverse with *ca*. 630 species. Scaly tree ferns consistently fall into four groups, with *Sphaeropteris* sister to the other three, *Cyathea*, *Alsophila* and *Gymnosphaera* (Korall et al., 2006; Korall et al., 2007; Korall & Pryer, 2014). All four groups occur in both America and Australasia, whereas only *Alsophila* and *Gymnosphaera* are represented in Africa (Korall & Pryer, 2014).

Cyatheaceae originated in the Late Jurassic in either South America or Australasia (Gondwana) according to Korall & Pryer (2014). Following a range expansion, the ancestral distribution of the marginate-scaled clade (*Cyathea*+*Alsophila*+*Gymnosphaera*) included both continents, whereas *Sphaeropteris* is reconstructed as having its origin only in Australasia (Korall & Pryer, 2014). Within the marginate-scaled clade, reconstructions of early divergences are hampered by the unresolved relationships among the *Alsophila*, *Cyathea* and *Gymnosphaera* lineages. Nevertheless, it is clear that the occurrence of the *Cyathea* and *Sphaeropteris* lineages in South America may be linked to vicariance related to the break-up of Gondwana, whereas transoceanic dispersal needs to be inferred for the range shifts seen in *Alsophila* and *Gymnosphaera* (Korall & Pryer, 2014). Although the occurrence of Cyatheaceae in the fossil record supports the South American origin of certain lineages, its presence in the fossil record indicates that these old lineages also occurred in Mesoamerica and North America. A *Cyathea* fossil from Vancouver Island is known from the lower Cretaceous, in the Barremian (Smith, Rothwell & Stockey, 2003), and fossil spores of *Alsophila* and *Cyathea* were reported from the Pliocene Gatun Formation of Panama (Graham, 1991),*Cyathea* and *Dicksonia* from Paraje Solo, Veracruz at the upper Miocene (Graham, 1991) and *Cyathea* from Pislepamba, Bolivia, at the Miocene-Pliocene boundary (6.7 Ma) in the lower limits of cloud forest-tropical forest transition (Graham, Gregory-Wodzicki & Wright, 2001). High percentage of *Oreomunnea* pollen grains and abundant spores of *Cyathea* and *Alsophila* indicate a paleoenvironment (Lower Miocene or Middle Miocene) dominated mostly by *Oreomunnea* forest growing in a coastal montane area of relatively cool and very wet climate in sedimentary rocks of northern Chiapas, near limits of Tabasco and Veracruz (Palacios-Chávez & Rzedowski, 1993).

Currently, tree ferns are mostly restricted to regions in which minimum temperatures rarely drop below freezing and rainfall is high and evenly distributed around the year (Watkins, Mack & Mulkey, 2007; Bystriakova, Schneider & Coomes, 2011). Cloud forests of tropical America harbor most of the species of American Cyatheales, mainly in Mesoamerica, the northern Andes, southeastern Brazil and the Antilles (Arens & Sánchez-Baracaldo, 1998; Arens, 2001; Kluge & Kessler, 2001; Ramírez-Barahona, Luna-Vega & Tejero-Díez, 2011). In Mexico, the distribution of most scaly tree ferns is limited almost entirely to the cloud forest, locally shaped by light sensitivity (Riaño & Briones, 2013). According to Bystriakova, Schneider & Coomes (2011) the strongest limiting factor on current worldwide distributions of scaly tree fern sporophytes is annual precipitation, which is consistent with data on gametophytes’ little tolerance to desiccation (Watkins et al., 2009). However, the current distribution of tree ferns in cloud forests and evolution of their climatic niche is also influenced by geography and historical factors (Bystriakova, Schneider & Coomes, 2011) and Quaternary climatic fluctuations (Ramírez-Barahona & Eguiarte, 2014) that have shaped the complex evolutionary history of these forests, in which a considerable range expansion and descent into lower elevations occurred during the Last Glacial Maximum (LGM; 20,000 years ago) (Graham, 1999; Still, Foster & Schneider, 1999; Caballero et al., 2010; Ramírez-Barahona & Eguiarte, 2014).

Despite the contradictory paleoecological information for reconstruction of past climate changes in the Neotropics (e.g., Ramírez-Barahona & Eguiarte, 2013), recent phylogeographic data suggest that cloud forest species persisted in these forests (Haffer, 1969; Ornelas & González, 2014) or expanded to the lowlands during glacial cycles and its distribution then contracted allopatrically during the interglacials (e.g., Ramírez-Barahona & Eguiarte, 2014; Ornelas & Rodríguez-Gómez, 2015; Ornelas et al., 2016). Alternatively, the evolutionary history of tree ferns reflects patterns of long-term persistence in the Mesoamerican cloud forests. Here we combine phylogenetic and ancestral area reconstruction and divergence time estimates with data for phenotypic traits (environmental space) of tree fern species to make inferences about evolutionary processes in deep time (Harmon et al., 2010; Mahler et al., 2010; O’meara, 2012). Specifically, we used phylogenetic Bayesian inference, estimates of divergence times, geographic distribution, and altitudinal and climatic data to investigate the ages of northern Mesoamerican tree ferns, and ask whether the ancestral area and ancestral elevation shifts of these tree ferns were linked to climatic events and historical climatic preferences and whether tree ferns history reflects patterns of long-term cloud forest persistence across northern Mesoamerica. Understanding how past climate change has affected the evolutionary history of tree ferns has future conservation implications considering their climate sensitivity, fragmented distribution, and current vulnerability of cloud forest communities worldwide.

## Methods

### Field study permissions

Our samples were collected in the living collections of the botanical garden “Francisco Javier Clavijero” and adjacent protected area of the Instituto de Ecología, A.C. (INECOL). We had access to the material under the terms of the scientific permit VER-FLO-228-09-09. The Mexican species of Cyatheaceae are under special protection (Norma Oficial Mexicana, NOM-059-ECOL-2010, Secretaría de Medio Ambiente y Recursos Naturales, Diario Oficial de la Federación 30 December 2010, Mexico, DF) and the botanical garden holds several individuals of every collected taxon. While the field collection involves endangered and protected species, no specific permits are required for field studies such as the one described here. Leaf tissue samples were obtained from the plant species reported here with no further manipulation.

### Sampling and field procedures

A total of 109 terminal taxa were included in the phylogenetic analyses, comprising 98 samples of representative taxa in the order Cyatheales, nine taxa of Polypodiales used as outgroups, and *Azolla mexicana* and *Marsilea mexicana* (Salviniales) selected as functional outgroups. Vouchers and GenBank accession numbers are provided in Table S1. The sampling covered tree fern species of eastern cloud forests in Mesoamerica and South America, representatives of major tree fern clades and Cyatheaceae as retrieved by previous phylogenetic studies (Korall et al., 2006; Korall et al., 2007), and representatives of polypod and heterosporous ferns as outgroups (Korall et al., 2006; Pryer et al., 2004).

### DNA extraction, amplification and sequencing

DNA was isolated using either a modified 2⋅CTAB method (Cota-Sánchez, Remarchuk & Ubayasena, 2006) or the DNeasy Plant MiniKit (Qiagen, Valencia, CA, USA) following the manufacturer’s instructions. Three genes (*atpA*, *atpB*, *rbcL*) and an intergenic spacer (*accDrbcL*) from the plastid genome were amplified using the polymerase chain reaction (PCR), following standard protocols. We sequenced the *rbcL* gene using the primers ESrbcL1F, ES645F, ES663R and ES1361R (Korall et al., 2006), *atpA* with primers ESATPF415F, ESATPA787F, ESATPA823R, ESATPA283F and ESTRNR46F (Korall et al., 2006), *atpB* with primers ATPB672F, ATPB1163F, ATPB1419F, ATPB1592R (Wolf, 1997), ATPB609R and ATPE384R (Pryer et al., 2004), and for *accD-rbcL* using primers RBCL1187Fa (Korall et al., 2006), ACCDHIF4 (Ebihara et al., 2003), ACCD887R (Korall et al., 2007) and ACCD816Ra (Ebihara et al., 2003). PCR products were purified with QIAquick columns (Qiagen) or ExoSAP-IT (Affymetrix, Santa Clara, CA, USA), sequenced with the TaqBigDye Terminator Cycle Sequencing kit (Perkin Elmer Applied Biosystems, Foster City, CA, USA) and processed on a 310ABI DNA sequencer (Perkin Elmer Applied Biosystems, Foster City, CA, USA). The sequences were edited in Sequencher v4.1 (Gene Codes, Ann Arbor, MI, USA) and aligned manually in Phyde (Müller, Müller & Quandt, 2010).

### Phylogenetic analyses

Bayesian Inference analysis was used to infer the phylogeny of plastid DNA (*rbcL, atpA, atpB, accD-rbcL*). The model of molecular evolution was determined in jModelTest v2.1.4 (Posada, 2008) using the Akaike Information Criterion (AIC) and default search values for every plastid marker. The best-fit models obtained were: GTR + I + G (*rbcL*), GTR + I + G (*atpA*), GTR + I + G (*atpB*), GTR + G (*accD-rbcL*). The Bayesian Markov chain Monte Carlo (BMCMC) was performed using MrBAYES v3.1.2 (Huelsenbeck & Ronquist, 2001) and the CIPRES Science Gateway (Miller, Pfeiffer & Schwartz, 2010). Analyses were carried out with two separate chains for each run (with three hot and one cold chains), running 10 million generations, sampling a tree every 1,000 generations. Stationarity was determined based on the convergence of likelihood scores, and sample points generated prior to stationarity were eliminated as burn-in (25%). The posterior probabilities (PP) of the clades were determined by a 50% majority consensus of the trees retained.

### Molecular dating analyses

Divergence times of Cyatheaceae and the order Cyatheales were estimated using BEAST v1.8.2 (Drummond & Rambaut, 2007). Analyses were run for 15 million generations, with parameter sampling every 1,000 generations and a burn-in of 10%, using an uncorrelated lognormal clock model, a Yule tree prior, and a random starting tree. The GTR + I + G evolutionary model was selected, given that it is the closest model to those calculated in jModelTest v0.1.1 (Posada, 2008). Three independent runs with a single data partition were performed and sampled values were jointly analyzed for convergence. Markov chain Monte Carlo (MCMC) chain mixing and convergence between runs were assessed by visual inspection of the likelihood effective sample size (ESS), traces and Bayesian density plots using TRACER v1.6 (Rambaut et al., 2014). For the combined runs, ESS for most of estimated parameters was >1,000. After removing the burn-in, the three runs were combined to obtain a maximum clade credibility tree with mean divergence times using TreeAnnotator (Drummond & Rambaut, 2007). The single tree was visualized with FIGTREE v1.5.4 (http://tree.bio.ed.ac.uk/software/figtree/).

Six calibration points based on fossil evidence were used in the dating analysis. These fossils have previously been used in phylogenetic analyses of ferns in general and scaly tree ferns in particular (Pryer et al., 2004; Korall & Pryer, 2014; Ramírez-Barahona, Barrera-Redondo & Eguiarte, 2016). The prior ages for the corresponding nodes were obtained from lognormal distributions with mean = 1, with an offset = estimated age of fossil, and standard deviation = 1 (Gradstein, Ogg & Smith, 2004). This placed the bulk of the distribution close to the absolute age assigned to each fossil, but allowed the possibility of very old ages (Gradstein, Ogg & Smith, 2004). For each fossil, absolute ages were set to the uppermost boundary of the stratigraphic interval to which the fossil could be assigned.

The six fossil used in the analysis (1) *Cyathocaulis* fossils (Upper Jurassic, 145.5 Ma) used to calibrate the most recent common ancestor of Cyatheaceae and its sister group Dicksoniaceae (Lantz, Rothwell & Stockey, 1999; Pryer et al., 2004); (2) the first appearance of fossils belonging to Kuylisporites mirabilis (Upper Cretaceous, 93.6 Ma) assigned to the most recent common ancestor of Cyatheaceae (Mohr & Lazarus, 1994; Korall & Pryer, 2014); (3) we considered fossils of *K*. *waterbolkii* as members of the stem lineage of *Cyathea* and its first appearance (Lower Eocene, 48.6 Ma) to calibrate the most recent common ancestor of *Cyathea* (Mohr & Lazarus, 1994); (4) the age of *Conantiopteris* (Aptian, Lower Cretaceous, 112 Ma) assigned to the most recent common ancestor of Dicksoniaceae (Lantz, Rothwell & Stockey, 1999); (5) age of fossils belonging to *Regnellites nagashimae* (Berriasian, Lower Cretaceous, 137 Ma) used to calibrate the divergence between Marsileaceae and Salviniaceae (Pryer et al., 2004); and (6) the age of fossils of *Athyrium* species from Northern China (Neocomian, Lower Cretaceous, 121 Ma) assigned to the most recent common ancestor of the Polypodiaceae (Chen, Deng & Sun, 1997; Skog, 2001; Pryer et al., 2004).

### Ancestral area and altitudinal reconstruction

We reconstructed ancestral geographic ranges using Bayesian methods with BBM (Bayesian Binary MCMC) analyses implemented in RASP v2.1b (Yu, Harris & He, 2010; Yu et al., 2015). These methods accommodate phylogenetic uncertainty by averaging the ancestral reconstructions over a sample of user-supplied trees. The 20,002 post-burn-in trees from the Bayesian Inference analyses using MrBAYES were input into RASP to estimate the probabilities of ancestral areas at each node on the condensed tree. We coded each of the Cyatheales and outgroup species in the data set as occurring in Mesoamerica (A), South America (B), Caribe (C), Africa (D), Asia and Pacific Islands (E), and Australia (F) (Table S2). Taxa that have a substantial distribution on either one side or the other of the Isthmus of Panama were coded as polymorphic (AB). The ancestral areas of terminal species representing outgroup taxa were scored for all portions of the genus or clade range. The maximum number of areas in ancestral ranges was constrained to six and the ancestral areas for nodes visualized on the condensed tree. For the BBM analyses we set a null distribution for the ancestral range of the root of the tree, and we ran ten MCMC chains simultaneously for 5-millions generations and the reconstructed state was sampled every 1,000 generations. The fixed model JC + G (Jukes-Cantor + Gamma) was used for BBM analysis with a null root distribution. We also reconstructed the ancestral altitudinal ranges based on the average altitude of each species (meters above sea level) and each species scored as one of three categories: (A) 0–1,000, (B) 1,001–2,000 and (C) 2,001–3,000 (Table  S2).

### Ancestral climatic and altitudinal preferences

To illustrate the climatic and altitudinal preferences evolution of tree ferns, a total of 20,782 occurrence data for the 109 terminal taxa were assembled from published taxonomic studies and complemented with data from three online databases (Missouri Botanical Garden’s Tropicos, http://www.tropicos.org); Global Biodiversity Information Facility, http://www.gbif.org; Red Mundial de Información sobre Biodiversidad of the Comisión Nacional para el Conocimiento y Uso de la Biodiversidad, (http://www.conabio.gob.mx/remib/doctos/remib_esp.html). Occurrence data were checked against systematic and monographic studies, thereby eliminating doubtful or unverified records. Finally, we restricted the data sets to unique records for the analyses, leaving 15,508 unique presence records for the full data set, 14,556 for tree ferns and 9,728 exclusive to Cyatheaceae. Present-day temperature and precipitation data (BIO1–BIO19 variables) were drawn as climate layers from the WorldClim database at a spatial resolution of *ca.* at 1 km^2^ in each raster (Hijmans et al., 2005). To overcome the multicollinearity problem among all variables, we performed principal components analysis (PCA) in Stata 11 (StataCorp LP, College Station, TX, USA) using the correlation matrix. We then ran correlation analysis to eliminate correlated environmental variables using the program PAST v2.12 (Hammer, Harper & Ryan, 2001), and for each pair of correlated variables we selected the ones with the highest loadings on each of the first three PC components (80% of the total variance). The resulting variables included BIO1 (annual mean temperature), BIO4 (temperature seasonality), BIO12 (annual precipitation), and BIO15 (precipitation seasonality). The average of these variables for each species was calculated and considered for mapping trait evolution in our tree fern phylogeny.

We also used ancestral character estimation to visualize historical character states for a continuous trait along the branches of a tree (Revell, 2013). By mapping the point estimates of ancestral states along the branches of the tree, this method ignores the uncertainty associated with ancestral character estimation of continuous climatic traits. Revell (2013) proposed a new method for visualizing ancestral state uncertainty using a type of projection of the tree into morphospace called a ‘traitgram’. Traitgrams arrange species along a continuous trait axis (the *x* axis) and connect them with their underlying phylogenetic tree (the *y* axis), allowing the visualization of trait evolution by having tips of a phylogeny positioned along a trait axis with internal nodes positioned according to ancestor trait reconstruction (Hammer, Harper & Ryan, 2001). The vertical position of internal nodes and branches are computed via ancestral character estimation using likelihood. The ancestral trait reconstruction was performed in R v2.15.2 (R Development Core Team, 2012) using the Phytools package (Revell, 2012). The average of the altitude ranges for each species was also calculated (Table S2) and considered as a mapping trait on our tree fern phylogeny.

To determine whether the resulting groups of tree ferns (*n* = 14, 556 occurrence records) and Cyatheaceae (*n* = 9, 728 occurrence records) are separated by environmental space (e-space) and whether lineages may have diverged with respect to e-space, we performed a principal components analysis (PCA) with varimax rotation (method: covariance matrix) to reduce intercorrelated temperature and precipitation to a smaller system of uncorrelated, independent variables. We examined principal components (PC) loadings to interpret the biological meaning of each PC. Differences in PC scores between groups of tree ferns (*Alsophila*, *Calochlaena*, *Cibotium*, *Culcita*, *Cyathea*, *Dicksonia*, *Lophosoria*, *Gymnosphaera*, *Loxoma*, *Metaxya*, *Plagiogyria*, *Sphaeropteris*, *Thyrsopteris*) or Cyatheaceae (*Alsophila*, *Cyathea*, *Sphaeropteris*, *Gymnosphaera*) were tested with a multiple analysis of variance (MANOVA) followed by one-way ANOVAs using group as a fixed factor and the resulting PC scores as dependent variables. Analyses were carried out in SPSS Statistics for Mac v17.0 (SPSS Inc., 2008).

## Results

### Phylogenetic analyses

**Figure 1:**
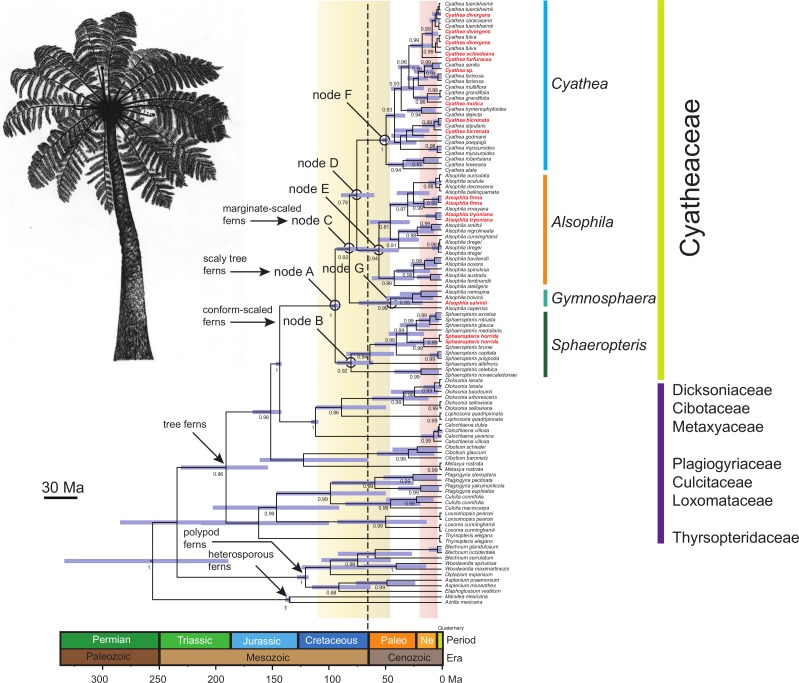
Chronogram of the Cyatheaceae and other tree fern lineages based on the calibration method with a Yule speciation model for the combined plastid DNA (*rbcL, atpA, atpB, accD-rbcl*) complete dataset. For selected nodes, 95% Highest Posterior Density (HPD) intervals, indicated here by purple bars, and other inferred divergence estimates are further summarized in the main text. These nodes all have posterior probabilities of 0.80–1.0. The root of the tree was calibrated using the age of the Core Leptosporangiates (307.9 ± 8.65 Ma), the divergence time estimated to water ferns Salviniales (161.59 ± 13.6 Ma), Polypodiales (181.87 ± 9.48), Cyatheales (191.4 ± 11.18), the divergence time between *Cibotium* (Dicksoniaceae) and Cyatheaceae (147.5 ± 1.7) and the age of Cyatheaceae crown clade (94.75 ± 0.6). Ages in geological time are shown at the base of the tree. The vertical yellow column indicates the time span of the Cretaceous–Paleogene (K–Pg) boundary and the pink column indicates the time span from the Miocene and Pliocene of the Tertiary flora relicts in Mesoamerica. The vertical dotted line indicates the K–Pg boundary (65 Ma). The boundary marks the end of the Cretaceous Period, which is the last period of the Mesozoic Era, and marks the beginning of the Paleogene Period of the Cenozoic Era. The boundary is associated with the Cretaceous-Paleogene mass extinction event, which is considered to be the demise of the non-avian dinosaurs, in addition to a majority of the world’s Mesozoic species. In bold and red are shown cloud forest-adapted tree fern species in Mesoamerica.

The phylogenetic analysis using Bayesian inference (BI) yielded the same general topology obtained for tree ferns in previous studies (Korall et al., 2006; Korall et al., 2007; Pryer et al., 2004; see Fig. S1), divided into three major sections: (1) “core tree ferns” *sensu* Korall et al. (2006) Cyatheaceae, Dicksoniaceae, Metaxyaceae, and Cibotaceae, (2) Culcitaceae + Plagiogyriaceae + Loxomataceae, and (3) Thyrsopteridaceae. How these four groups are related to one other is, however, weakly supported (Fig. S1). Only relationships with a posterior probability (PP) of >0.80 are discussed below, unless otherwise stated. Within Cyatheaceae, the BI showed a highly supported sister relationship between the conform-scaled ferns (*Sphaeropteris*) and the marginate-scaled ferns clade (*Gymnosphaera*, *Alsophila* and *Cyathea*; PP = 0.82; Fig. S1). Because the resulting relationships within Cyatheaceae are congruent with previous studies (Korall et al., 2006; Korall et al., 2007; Pryer et al., 2004), we consider the resulting BI tree topology to be a reasonable estimate. The topology resulting from the MrBAYES analysis (Fig. S1) was also congruent with the BEAST analysis (Fig. 1).

### Molecular dating analyses

Relationships between tree fern species estimated in BEAST (Fig. 1) strongly supported common ancestry for the Cyatheaceae (PP = 1). Results from BEAST suggest that Cyatheaceae diverged from its sister lineage 147 Ma in the Late Jurassic, with the crown group originating 96.9 Ma in the Mid-Cretaceous (95% HPD 100.9–93.6 Ma; node A, Fig. 1). Within Cyatheaceae, the crown group of *Sphaeropteris* (conform-scaled ferns) dates back to 82.4 Ma (95% HPD 95.5–62.2 Ma, node B), the crown group age of the marginate-scaled clade is estimated to be 83.9 Ma (Late Cretaceous; 95% HPD 95.5–67.6 Ma; node C) and the *AlsophilaCyathea* split 77.2 Ma (95% HPD 91.6–61.2 Ma; node D, Fig. 1), with *Alsophila*, *Cyathea* and *Gymnosphaera* having crown group origins around the Paleocene-Eocene boundary (57 Ma, 95% HPD 75.8–38.7 Ma; 50.6 Ma, 95% HPD 54.3–48.6 Ma; 46.4 Ma, 95% HPD 75.5–17.3 Ma nodes E, F and G, Fig. 1). Most species divergence within the marginate-scaled clade occurred from the Miocene to the Pliocene.

### Ancestral area and altitudinal reconstruction

**Figure 2:**
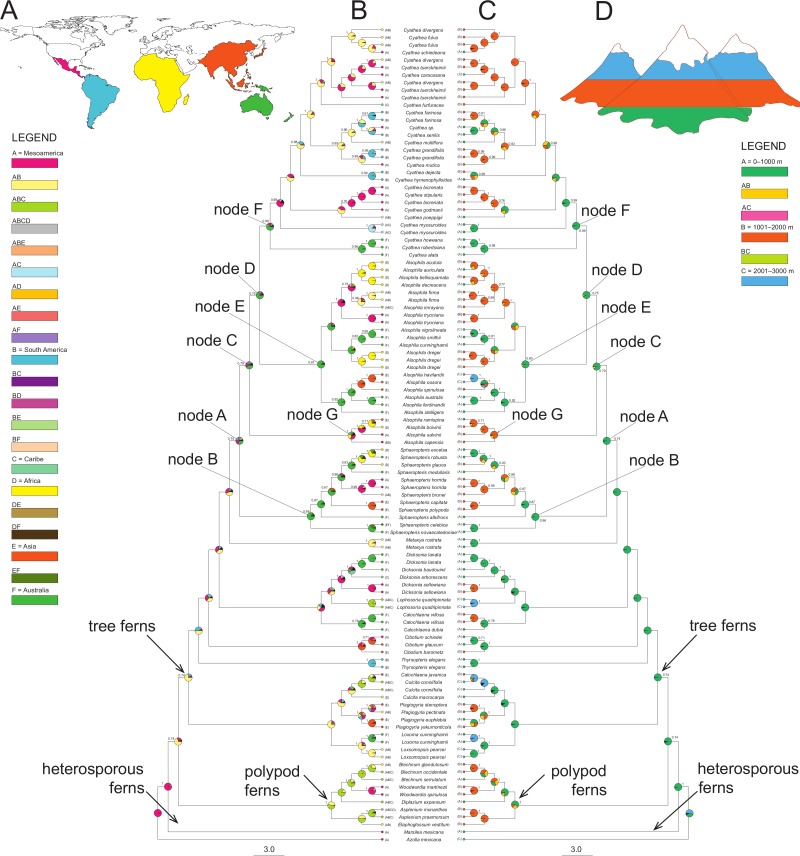
A summary of the BBM (Bayesian Binary MCMC) analyses implemented in RASP of the core Cyatheaceae and allies. (A) Biogeographical regions used in the S-DIVA analysis: Mesoamerica (A), South America (B), Caribe (C), Africa (D), Asia and Pacific Islands (E), and/or Australia (F). (B) The tree is a majority-rule consensus of 20,002 trees derived from Bayesian inference analysis of the *rbcL, atpA, atpB,* and *accD-rbcL* plastid DNA sequences from tree ferns. This tree was highly consistent with those inferred using Bayesian inference. Current geographical range for each taxon, as delimited in (a), is drawn on the terminal lineages before each taxon’s name. Pie charts at internal nodes represent the marginal probabilities for each alternative ancestral area. These probabilities account for the phylogenetic uncertainty (black color in pie charts) in the rest of the tree and the biogeographical uncertainty (multiple equally parsimonious reconstructions) at each node. (C) Elevational shifts along the phylogeny of tree ferns according to the BMM analysis. Altitudinal ranges of each species based on the average altitude of each species (meters above sea level) and coded as: 0–1,000 (A), 1,001–2,000 (B) and 2,001–3,000 (C). (D) Altitudinal representation code used in (C).

The BBM (MCMC) reconstructions required at least six dispersal events to explain the present-day distribution when the maximum number of areas at each node was not restricted (Fig. 2). RASP favors an ancestral distribution in Australia (code F) for most of the clades (32.6% node A, 83.5% node B, 43.4% node C, 69.9% node D, 66.5% node E and 66.55% for node F), with the exception of node G (*Gymnosphaera*; Fig. 2) that was ambiguous (Mesoamerica, code A, 30.5%; Africa, code D, 27.2%; South America, code B, 15.6%).

Elevational shifts are scattered along the phylogeny of tree ferns (Fig. 2). According to reconstructed ancestral elevation, the most recent common ancestor of Cyatheaceae was a lower montane forest species (A, 97.8%, node A). Reconstruction of ancestral altitudinal preferences shown in Fig. 2 indicates elevated probability of shifts from low-elevation habitats to middle-to-high elevation habitats (nodes A to F). Middle-to-high montane forests appear as the most probable ancestral habitat throughout the next internal nodes in our phylogeny, suggesting that habitats at higher elevations (cloud forests) may be the result of independent colonization events.

### Ancestral climatic and altitudinal preferences

**Figure 3:**
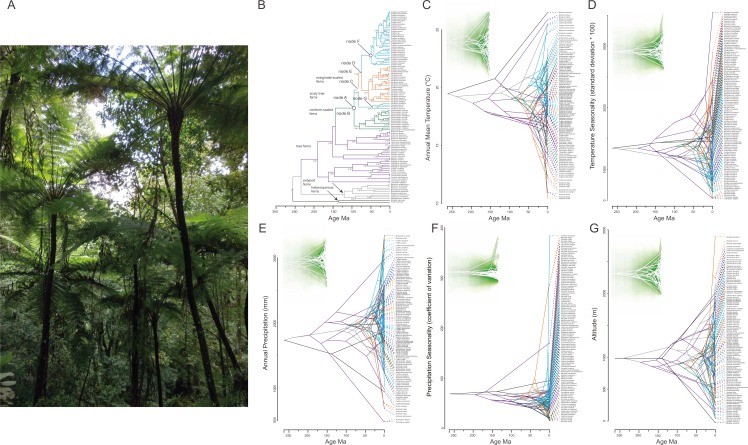
Phylogenetic tree for 98 tree fern species using Bayesian inference (A) and traitgrams depicting ancestral climatic preferences and shifts at (B, H) annual mean temperature (BIO1), (C, I) temperature seasonality (BIO4), (D, J) annual precipitation (BIO12), (E, K) precipitation seasonality (BIO15) and (F, L) altitude (*x* axes) and their underlying phylogenetic relatedness (time from the root; *y* axis). The high level of branches crossing shows convergent evolution across clades and weak phylogenetic signal. Colors correspond to the clades in the phylogenetic tree shown in (A). Time of divergence is based on our estimations. On the upper side of trees uncertainty is shown via increasing transparency of the plotted lines around the point estimates with the entire range showing the 95% confidence interval (H–L). Highest transparency is seen at early divergence time in all variables. (G) *Cyathea bicrenata* tree ferns in the surroundings of Xalapa, Veracruz (photo by Etelvina Gándara).

The evolution of climatic tolerances becomes explicit when the history of niche occupancy is reconstructed (Fig. 3). Divergent evolution (within clades) and convergent evolution (among clades) with respect to mean annual temperature (BIO1), temperature seasonality (BIO4), annual precipitation (BIO12), precipitation seasonality (BIO15) and altitude is apparent, causing the lines connecting putative ancestors with their descendants to cross (Fig. 3).

Contemporary climate variation was significantly different among groups of tree ferns (Wilks’ Lambda = 0.361, *F*_22,29086_ = 877.09, *P* < 0.0001; Fig. S2). The PCA reduced measures of climatic variation (BIO 1–19 variables) to two PC components that explained 59.8% of the total variance. The first factor (PC1, 34.4%) was largely a measure of precipitation conditions, and the second factor (PC2, 24.7%) was mainly determined by temperature measures (Table S3). Univariate ANOVAs of both PC1 and PC2 scores showed significant differences between groups of tree ferns (Table S3). Among groups of Cyatheaceae, climate variation was also significantly different (Wilks’ Lambda = 0.500, *F*_4,19448_ = 2011.03, *P* < 0.0001; Fig. S2). The PCA reduced measures of climatic variation (BIO 1–19 variables) to two PC components that explained 63.2% of the total variance. The first factor (PC1, 32.4%) was largely a measure of precipitation conditions, and the second factor (PC2, 30.8%) was mainly determined by temperature measures (Table S3). Univariate ANOVAs of both PC1 and PC2 scores showed significant differences between groups of Cyatheaceae (Table S3). The ecological gradient in both data sets is seen in the highest (±0.8) factor loadings of the BIOCLIM variables (Table S3): the most positive are BIO12 (annual precipitation), BIO16 (precipitation of wettest quarter) and BIO3 (isothermality), and the most negative are BIO4 (temperature seasonality) and BIO7 (temperature annual range).

## Discussion

### Time of divergence and ancestral area reconstruction

The phylogenetic relationships retrieved here for Cyatheaceae are mostly congruent with previous phylogenetic studies (Korall et al., 2006; Korall et al., 2007); species in *Cyathea*, *Alsophila Gymnosphaera* and *Sphaeropteris* were retrieved in their respective clades, coinciding with Korall et al. (2006); Korall et al. (2007). Note that *Gymnosphaera* species are presented here as *Alsophila* because of unresolved nomenclatural problems. Divergence times of the four groups were mostly in the Mid-Cretaceous, agreeing with earlier estimation of divergence times (Pryer et al., 2004). Moreover, our divergence time estimates coincide with recent estimations for the scaly ferns in which the *Alsophila, Cyathea* and *Sphaeropteris* clades diverged during the Paleogene, approximately 50–30 Ma (Korall & Pryer, 2014). However, splits for species found in the Mesoamerican cloud forests (Fig. 1), are more recent, from the Neogene to the Quaternary. This corresponds with other groups in the gymnosperms such as cycads and *Ephedra*, in which ancient lineages underwent a re-diversification beginning in the Miocene into the Pleistocene (Nagalingum et al., 2011; Loera, Ickert-Bond & Sosa, 2015).

According to Pryer et al. (2004) Cyatheaceae originated in Gondwana. Fossils of *Cyathea* recorded in Vancouver Island, in the Barremian (Smith, Rothwell & Stockey, 2003) and fossil spores of this genus in South America in the Miocene-Pliocene boundary (Graham, 1991) do not fully support a South American origin. Based on the analysis of fourteen palynofloras in a number of localities throughout the Neotropics, Graham (1991) and Graham (1999) and Graham, Gregory-Wodzicki & Wright (2001) suggested a dispersal pattern in which temperate elements of the vegetation was from north-to-south and early-to-late Miocene, as previously suggested by Tryon (1971) for the *Sphaeropteris horrida* complex.

Our results identified that this family originated in Australia when it formed part of Gondwana. The ancestral reconstruction favored by RASP for most of the nodes in the phylogeny does not correspond to the distribution of continental plates and of areas above-sea-level (i.e., cloud forests) at the times inferred to these nodes by BEAST analysis. Furthermore, our results suggest that independent colonization events occurred from Australia to the New World at different times, the most recent during the Paleogene in the transition from Eocene-Oligocene. Biogeographic histories in ferns are commonly complex because the ancestors of early diverging clades might have dispersed long distances repeatedly or instead have greatly expanded distributions as indicated by our results. For instance, the biogeographic history of Dryopteridaceae is complicated, several disjunctions related to independent long-distance dispersal events over the Indian and the Pacific Ocean during the Neogene in *Polystichum* were identified ( Le Péchon et al., 2016), and in the Lastriopsids multiple interchanges were inferred between Australia and South America during the Oligocene and Eocene when these two areas were still connected by Antarctica (Labiak et al., 2014). In scaly tree ferns from the Galápagos and Cocos islands independent colonization events were also detected from separate sources in mainland America (Kao et al., 2015).

However, our ancestral reconstruction should be interpreted with caution until a larger number of cloud forest-adapted species has been included. Cyatheaceae is a family comprising approximately 630 species, from which *ca*. 200 are Neotropical and the majority belong to *Cyathea* (Lehnert, 2006a; Lehnert, 2006b; Lehnert, 2011; Ramírez-Barahona, Barrera-Redondo & Eguiarte, 2016), with a distribution range of most species centered in the eastern slopes of the Andes in South America and eight species distributed in the Western Pacific (Lehnert, 2006a; Lehnert, 2006b). Based on our sampling of *Cyathea* species, contradictory data on the distribution of fossil Cyatheaceae in the New World, and the Australian ancestral reconstruction for critical nodes in the phylogeny of this group, we suggest that further research including more *Cyathea* species might bring understanding on the biogeographic history of this group of tree ferns, particularly to assess the combined effects of plate tectonics from the Cretaceous onwards and dispersal/range shifts from climate changes on drifting ancestors from the lowlands to colonize cloud forests at higher  elevations.

### Climatic and altitudinal distribution of tree ferns

Bystriakova, Schneider & Coomes (2011) reconstructed the ancestral climatic niches of tree fern species by maximum likelihood and least-squares analyses, and identified annual precipitation as one of the climatic variables limiting tree fern distribution and a wet tropical niche for the common ancestor of scaly tree ferns. Their analyses indicated that tree ferns are restricted to regions in which minimum temperatures rarely drop below freezing and where rainfall is high and evenly distributed around the year. Warm and wet environments characterized by low seasonality provide the best opportunity for the majority of ferns to become established (Holttum, 1985; Farrar et al., 2008). Therefore, their results suggesting that the distributions of tree fern sporophytes strongly limited by annual precipitation is consistent with the current interpretation of gametophyte ecological observations (Bystriakova, Schneider & Coomes, 2011). Thus, we used distributions of extant descendants to assess climate niches of ancestors assuming that tree ferns are only able to inhabit in warm and wet habitats as suggested by Hinojosa et al. (2015). Nonetheless, the phylogenetic reconstruction methods used here to infer ancestral distributions and climatic niches based on present distributions of extant taxa are likely to lead to erroneous results when climatic requirements of ancestors differ from their extant descendants, or when much extinction has occurred (Hinojosa et al., 2015). Although some evidence of variation in extinction rates has been recently reported among three high-diversity clades (i.e., *Sphaeropteris* clade, *A. australis* clade and *C. multiflora* clade; a more densely sampled phylogeny would be needed to fully test hypotheses related to clade-specific extinction rates.

Our study focused on the biogeography of Mesoamerican tree ferns. Interestingly, our analyses showed that several ancestors of tree ferns have invaded independently the Mesoamerican cloud forests. Regarding the directionality of niche evolution, reconstructed distribution of mean climatic tolerance more than maximum or minimum climate tolerance and divergent versus convergent niche evolution were inferred (see Knouft et al., 2006; Evans et al., 2009). Our results did not identify divergent niche evolution from the hypothesized ancestors of *Sphaeropteris* and *Alsophila* groups considering the four most significant climate variables and altitude. In *Cyathea* the exception was in the annual mean temperature, in which an incipient divergence, a change from colder to warmer temperatures was visualized (Fig. 3B). With regard to evolution of preferences for annual precipitation our results showed that the ancestral node for the Cyatheaceae and the internal nodes of *Alsophila*, *Cyathea* and *Sphaeropteris* are not divergent from their hypothesized ancestor. All groups inhabit environments with elevated annual precipitation (2,000–2,500 mm). Nonetheless, further reconstruction of ancestral climatic niches of species testing niche conservatism/divergence in a phylogenetic framework should include a more adequate representation of species including those capturing all ranges of environmental variation.

For elevational shifts we found *Sphaeropteris*, *Cyathea* and *Alsophila* species changing from low- to mid-elevations (Fig. 2B). These results partially coincide with Lehnert (2011) reporting that related species in *Cyathea* from Central America and the Andes share preferences for higher altitudes. For Mesoamerica, paleoecological data are controversial and scarce. However, indirect evidence of paleorecords suggests that in the Late Pleistocene climate changes were significant; climate in the mountain areas of eastern Mexico was humid during this period, however, at the boundary of Pleistocene/Holocene, aridification started (Metcalfe et al., 2000). Moreover, for the Last Glacial Maximum two hypothetical models related to the role that climate changes played on the distribution of montane vegetation in Mesoamerica have been proposed: the dry refugia model and the moist forests model (reviewed in Ramírez-Barahona & Eguiarte, 2013. In the dry refugia model, species are spatially displaced by aridity during the Last Glacial Maximum while in the moist forests model no major changes of humidity occurred but down-slope range expansion occurred due to temperature descent at higher elevations (Ramírez-Barahona & Eguiarte, 2013). Our results suggest that the decrease of humidity probably drove up-slope colonization from lowland populations, coinciding with our dating analyses that identified divergence events between cloud forest tree fern species and those from lowland tropical rainforests during these periods in clades *Sphaeropteris*, *Cyathea* and *Alsophila*.

### Evolution of cloud forest-adapted tree ferns in Mesoamerica

Complex evolutionary and biogeographic scenarios in the history of the Cyatheaceae tree ferns are clearly apparent from our results. Despite phylogenetic uncertainty, particularly concerning the tree fern species from cloud forests in Mesoamerica, our data show that these species do not represent a single radiation in cloud forests ecosystems. Rather they appear to represent independent events of adaptive colonization of cloud forests over time (convergent ecological evolution), whether derived from Mesoamerican sister species from the lowlands, and that they have been intermixed with tropical broadleaved-evergreen trees species since the Miocene (Miranda & Sharp, 1950; Sharp, 1951; Williams-Linera, 1997; Graham, 1999; Qian & Ricklefs, 2004; Ruiz-Sanchez & Ornelas, 2014). Sequence and time divergences based on plastid DNA suggest a recent Miocene to Pliocene evolution of *Alsophila* and *Cyathea* tree ferns in the cloud forests of Mesoamerica with respect to other members of Cyatheaceae (*Sphaeropteris*). Although there is phylogeographic evidence of genetic divergence and range shifts from lowland tropical forests to cloud forests for Mesoamerican cloud forest species (e.g., Ornelas et al., 2016), only few examples have documented repeated habitat shifts in relation to climate changes along the evolutionary history of a lineage. For instance, the diversification of *Bursera*, from the Miocene onwards, occurred during a period of enhanced aridity, with divergence times between *B. microphylla, B. hindsiana, B. laxiflora and B. fagaroides* and its corresponding closest relatives, and range shifts from seasonally dry tropical forest to xerophytic scrubs, estimated between 7.7 and 4.6 Ma (De-Nova et al., 2012).

Overall, our biogeographical analysis suggests that Cyatheaceae tree ferns have colonized the Mesoamerican cloud forests from the lowlands on several occasions (i.e., pseudocongruence) and that those that currently occur in cloud forests need to be included in further phylogeographic studies accompanied with species distribution modeling to test whether they are each other’s closest relatives. This lends further support for case-by-case exploration of the cloud forest-adapted tree ferns. A comparative phylogeographic analysis of Mesoamerican tree ferns adapted to cloud forests and their closest relatives in the lowlands will bring additional insights into to the observed pseudocongruence on their ancestral area reconstruction, levels of intraspecific genetic diversity, influence of geographic barriers, and the history of colonization events to higher elevation. Clearly, phylogeographical and environmental data of other cloud forest-adapted tree ferns are critically important to understand the tempo of species diversification and the role of climatic factors driving genetic differentiation and species distribution modeling will broaden our understanding on the importance of cloud forests in Mesoamerica in the evolution of tree ferns.

##  Supplemental Information

10.7717/peerj.2696/supp-1 Table S1 GenBank Species names and GenBank accession numbers for the specimens included in this study.

10.7717/peerj.2696/supp-2 Table S2 Altitudinal data

10.7717/peerj.2696/supp-3 Table S3 PCA

10.7717/peerj.2696/supp-4 Figure S1 MrBayes

10.7717/peerj.2696/supp-5 Figure S2 Climate comparisons

## Additional Information and Declarations

### Field Study Permissions

The following information was supplied relating to field study approvals (i.e., approving body and any reference numbers): The following information was supplied relating to field study approvals (i.e., approving body and any reference numbers): Secretaría de Medio Ambiente y Recursos Naturales, Instituto Nacional de Ecología, Dirección General de Vida Silvestre (permit number: Registro de Colección Científica VER-FLO-228-09-09).

### DNA Deposition

The following information was supplied regarding the deposition of DNA sequences: GenBank accession numbers are provided in Table S1.

### Data Availability

The following information was supplied regarding data availability: Sosa V. Ornelas JF, Ramírez-Barahona S, Gándara E. 2016. Historical reconstruction of climatic and elevation preferences and the evolution of cloud forest-adapted tree ferns in Mesoamerica. Dryad Digital Repository. DOI: 10.5061/dryad.709t8.
