# Supplementary material for: Historical reconstruction of climatic and elevation preferences and the evolution of cloud forest-adapted tree ferns in Mesoamerica
Source: PeerJ. 2016 Nov 16;4:e2696. doi: 10.7717/peerj.2696 (PMC5119233; doi:10.7717/peerj.2696)
Supplement: Table S1 — Species names and GenBank accession numbers for the specimens included in this study. [file peerj-04-2696-s001.docx]

**Table S1. Species names and GenBank accession numbers for the specimens included in this study.** In bold are shown cloud forest-adapted tree fern species in Mesoamerica.

|  | Species | *rbcL* | *atpA* | *atpB* | *accD-rbcL* |
| --- | --- | --- | --- | --- | --- |
| 1 | *Alsophila acutula* R.M. Tryon | AM497473 | -- | -- | AM495807 |
| 2 | *Alsophila auriculata* (Tardieu) M.T. Tryon | AM495730 | -- | -- | AM495812 |
| 3 | *Alsophila australis* R,Br, | AM177319 | AM176419 | -- | AM410453 |
| 4 | *Alsophila bellisquamata* (Bonap.) R.M. Tryon | AM495733 | -- | -- | AM495815 |
| 5 | *Alsophila boivinii* Mett. | KT235817 | -- | -- | -- |
| 6 | *Alsophila capensis* J.Sm. | AM177321 | AM176421 | AM176582 | AM410455 |
| 7 | *Alsophila cunninghamii* (Hook.f.) R.M.Tryon | AM410211 | -- | -- | AM410482 |
| 8 | *Alsophila decrescens* (Kuhn.) R.M. Tryon | -- | -- | -- | AM495840 |
| 9 | *Alsophila dregei* (Kunze) R.M.Tryon | AM495759 | EF463630 | EF463360 | AM495843 |
| 10 | *Alsophila dregei* (Kunze) R.M.Tryon | AM410194 | -- | -- | AM410454 |
| 11 | *Alsophila ferdinandii* R.M.Tryon | AM410204 | -- | -- | AM410476 |
| 12 | ***Alsophila firma* (Baker) D.S.Conant** | KX395653 | KX395679 | KX395695 | KX395666 |
| 13 | *Alsophila havilandii* (Baker) R.M.Tryon | AM410189 | FN667541 | FN667550 | AM410447 |
| 14 | *Alsophila imrayana* (Hook.) D.S.Conant | AM410202 | FN667542 | FN667551 | AM410469 |
| 15 | *Alsophila nigrolineata* (Holttum) R.M.Tryon | AM410206 | -- | -- | AM410478 |
| 16 | *Alsophila oosora* (Holttum) R.M.Tryon | AM410209 | -- | -- | AM410480 |
| 17 | *Alsophila ramispina* Hook. | AM177326 | EF463633 | AM176587 | AM410463 |
| 18 | ***Alsophila salvinii* Hook.** | AM410184 | EF463634 | EF463361 | AM410438 |
| 19 | *Alsophila smithii* Trevis. | AM410210 | -- | -- | AM410481 |
| 20 | *Alsophila spinulosa* (Hook.) R.M.Tryon | AM410212 | FJ556581 | AY796299 | AM410483 |
| 21 | *Alsophila stelligera* (Holttum) R.M.Tryon | AM410198 | EF463635 | EF463362 | AM410465 |
| 22 | ***Alsophila tryoniana* (Gastony) D.S.Conant** | AM410208 | -- | AM410271 | -- |
| 23 | ***Alsophila tryoniana* (Gastony) D.S.Conant** | KX395660 | KX395686 | KX395702 | KX395673 |
| 24 | *Asplenium monanthes* L. | AY300125 | EF463599 | EF463335 | -- |
| 25 | *Asplenium praemorsum* Sw. | KP985726 | EF463604 | EF463340 | -- |
| 26 | *Azolla mexicana* Schlecht. & Cham. | EF520923 | -- | EF520873 | -- |
| 27 | *Blechnum glandulosum* Wall. | AB040555 | -- | -- | -- |
| 28 | *Blechnum occidentale* L. | EU352296 | EF452080 | BOU93838 |  |
| 29 | *Blechnum serrulatum* Rich. | AY137671 | -- | -- | -- |
| 30 | *Calochlaena dubia* (R.Br.) M.D. Turner & R.A. White | U05615 | AM176427 | AY612690 | AM410496 |
| 31 | *Calochlaena javanica* (Blume) M.D.Turner & R.A.White | HQ334992 | -- | -- | -- |
| 32 | *Calochlaena villosa* (C.Chr.) G.B.Nair | CVU05912 | EF463658 | AM176588 | AM410497 |
| 33 | *Cibotium barometz* (L.) J.Sm. | CBU05610 | AM176429 | AM176589 | JX485694 |
| 34 | *Cibotium glaucum* (Sm.) Hook. & Arn. | CGU05913 | AM176431 | AM176591 | JX485696 |
| 35 | *Cibotium schiedei* Schltdl. & Cham. | AM177331 | EF463624 | AM176593 | JX485698 |
| 36 | *Culcita conniifolia* (Hook.) Maxon | CCU18648 | EF463625 | AM176595 | -- |
| 37 | *Culcita macrocarpa* C.Presl | AM177334 | AM176436 | AM176596 | -- |
| 38 | *Cyathea alata* Copel. | AM177335 | AM176437 | AM176597 | AM410436 |
| 39 | ***Cyathea bicrenata* Liebm.** | -- | -- | -- | KX395675 |
| 40 | *Cyathea caracasana* Domin | AM410223 | -- | -- | AM410493 |
| 41 | *Cyathea dejecta* (Baker) Christenh. | AB5747511 | -- | -- |  |
| 42 | ***Cyathea divergens* Kunze** | KX395654 | KX395680 | KX395696 | KX395667 |
| 43 | ***Cyathea divergens* Kunze** | KX395655 | KX395681 | KX395697 | KX395668 |
| 44 | ***Cyathea divergens* Kunze** | KX395656 | KX395682 | KX395698 | KX395669 |
| 45 | ***Cyathea divergens* Kunze** | KX395657 | KX395683 | KX395699 | KX395670 |
| 46 | *Cyathea farinosa* (H.Karst.) Domin | HM446787 | -- | -- | -- |
| 47 | ***Cyathea fulva* (M. Martens & Galeotti) Fée** | -- | -- | -- | KX395677 |
| 48 | ***Cyathea fulva* (M. Martens & Galeotti) Fée** | -- | -- | -- | KX395678 |
| 49 | ***Cyathea furfuracea* Baker** | AM410224 | -- | -- | AM410494 |
| 50 | *Cyathea godmanii* Domin |  |  |  |  |
| 51 | *Cyathea grandifolia* Willd. | AM177332 | AM176434 | AM176594 | AM410440 |
| 52 | *Cyathea grandifolia* Willd. | AM177332 | AM176434 | AM176594 | -- |
| 53 | *Cyathea howeana* Domin | AM410188 | FN667545 | FN667554 | AM410446 |
| 54 | *Cyathea hymenophylloides* (L.D.Gómez) Christenh. | AF101302 | -- | -- | -- |
| 55 | ***Cyathea miosuroides* Domin** | -- | -- | -- | KX395676 |
| 56 | *Cyathea multiflora* Sm. | AM410197 | EF463638 | EF463365 | AM410461 |
| 57 | ***Cyathea mutica* (Christ) Domin** | AM410220 | -- | -- | AM410490 |
| 58 | *Cyathea poeppigii* Domin | AM410201 | EF463640 | AF313553 | AM410468 |
| 59 | *Cyathea robertsiana* Domin | AM410216 | -- | -- | AM410486 |
| 60 | ***Cyathea schiedeana* Domin** | KX395659 | KX395685 | KX395701 | KX395672 |
| 61 | *Cyathea senilis* Domin | AM410203 | FN667546 | FN667555 | AM410473 |
| 62 | ***Cyathea* sp.** | KX395661 | KX395687 | KX395703 | -- |
| 63 | ***Cyathea* sp.** | KX395662 | KX395688 | KX395704 | -- |
| 64 | ***Cyathea* sp.** | KX395663 | KX395689 | KX395705 | -- |
| 65 | ***Cyathea* sp.** | -- | KX395690 | KX395706 | -- |
| 66 | ***Cyathea* sp.** | -- | KX395691 | -- | -- |
| 67 | ***Cyathea* sp.** | -- | KX395692 | -- |  |
| 68 | *Cyathea stipularis* Domin | AM410219 | -- | -- | AM410489 |
| 69 | *Cyathea tuerckeimii* Maxon | HM446787 | -- | -- | -- |
| 70 | *Cyathea tuerckeimii* Maxon | KR082866 | -- | -- | -- |
| 71 | *Cyathea tuerckeimii* Maxon | KR082865 | -- | -- | -- |
| 72 | *Dicksonia arborescens* L'Hér. | AM177340 | AM176443 | AM176602 | AM410499 |
| 73 | *Dicksonia baudouinii* E.Fourn. | EF469951 | -- | -- | -- |
| 74 | *Dicksonia lanata* Colenso | EF469950 | AM176446 | AM176605 | AM410500 |
| 75 | ***Dicksonia sellowiana* (Pr,) Hook.** | KX395658 | KX395684 | KX395700 | KX395671 |
| 76 | *Diplazium expansum* Willd. | KP985729 | -- | -- | -- |
| 77 | *Elaphoglossum vestitum* (Schltdl. & Cham.) Schott | KP985730 | -- | -- | -- |
| 78 | *Lophosoria quadripinnata* C.Chr.in Skottsb. | AF101303 | EF463660 | AY612701 | AM410505 |
| 79 | *Loxsoma cunninghamii* R.Br.; A.Cunn. | AY612679 | EF463779 | AY612702 | -- |
| 80 | *Loxsomopsis pearcei* (Baker) Maxon | AY612680 | EF463780 | AY612703 | -- |
| 81 | *Marsilea mexicana* A.Braun | HQ631081 |  |  |  |
| 82 | *Metaxya rostrata* C.Presl | AF317700 | DQ390569 | AY612705 | -- |
| 83 | *Metaxya rostrata* C.Presl | AM177346 | EF463791 | AM176610 | -- |
| 84 | *Plagiogyria euphlebia* (Kunze) Mett. | JF303965 | JF303986 | -- | -- |
| 85 | *Plagiogyria pectinata* (Liebm.) Lellinger | AM177347 | AM176455 | AM176614 |  |
| 86 | *Plagiogyria stenoptera* (Hance) Diels | AB574747 | -- | -- | -- |
| 87 | *Plagiogyria yakumonticola* Nakaike | AB574748 | -- | -- | -- |
| 88 | *Sphaeropteris albifrons* (Fourn.) R.M.Tryon | AM410214 | -- | -- | AM410484 |
| 89 | *Sphaeropteris brunei* (Christ) R.M. Tryon | AM177349 | AM176457 | AM176616 | AM410439 |
| 90 | *Sphaeropteris capitata* (Copel.) R.M.Tryon | AM410192 | EF463642 | EF463366 | AM410450 |
| 91 | *Sphaeropteris celebica* (Blume) R.M.Tryon | AM410195 | EF463643 | EF463367 | AM410456 |
| 92 | *Sphaeropteris excelsa* (R.Br. ex Endl.) R.M.Tryon | AM410213 | -- | -- | -- |
| 93 | *Sphaeropteris glauca* (Blume) R.M.Tryon | AM410193 | FN667548 | FN667557 | AM410451 |
| 94 | ***Sphaeropteris horrida* (Liebm.) R.M.Tryon** | KP985731 | EF463644 | EF463368 | AM410467 |
| 95 | *Sphaeropteris medullaris* Bernh. | AM177350 | AM176458 | AM176617 | AM410452 |
| 96 | *Sphaeropteris* *novae-caledoniae* (Mett.) R.M. Tryon | AM177351 | AM176459 | AM176618 | AM410474 |
| 97 | *Sphaeropteris polypoda* (Baker) R.M.Tryon | AM410191 | FN667549 | FN667558 | AM410449 |
| 98 | *Sphaeropteris robusta* (Watts) R.M.Tryon | AM410187 | EF463647 | EF463370 | AM410445 |
| 99 | *Thyrsopteris elegans* Kunze | AM177353 | EF463900 | AM176620 | HG422549 |
| 100 | *Woodwardia maximartinezii* Maxon ex Weath. | AY137661 | -- | -- | -- |
| 101 | *Woodwardia spinulosa* M.Martens & Galeotti | AY137668 | -- | -- | -- |
