# Supplementary material for: Historical reconstruction of climatic and elevation preferences and the evolution of cloud forest-adapted tree ferns in Mesoamerica
Source: PeerJ. 2016 Nov 16;4:e2696. doi: 10.7717/peerj.2696 (PMC5119233; doi:10.7717/peerj.2696)
Supplement: Table S2 [file peerj-04-2696-s002.doc]

**Table S2 Altitudinal data (mean ± SD, minimum, maximum) and distributional and altitudinal codes for RASP analyses.** In bold are shown cloud forest-adapted tree fern species in Mesoamerica.

| **Species** | **Altitude**  **Min** | **Altitude** | **Altitude Mean** | **Altitude** | **Distributional code** | **Altitudinal code** |
| --- | --- | --- | --- | --- | --- | --- |
|  |  | Max |  | SD |  |  |
| *Alsophila acutula* | 307 | 2236 | 1082.0625 | 378.1694395 | D | B |
| *Alsophila auriculata* | 471 | 1062 | 813.8333333 | 192.5808056 | D | A |
| *Alsophila australis* | 1 | 1314 | 470.8259619 | 335.4354101 | F | A |
| *Alsophila bellisquamata* | 17 | 1669 | 1107.633333 | 432.75154 | D | B |
| *Alsophila boivinii* | 607 | 1648 | 1114.853659 | 247.8163192 | D | B |
| *Alsophila capensis* | 340 | 1888 | 1215.055556 | 414.7941335 | BD | B |
| *Alsophila cunninghamii* | 8 | 1042 | 323.3806647 | 186.0408072 | F | A |
| *Alsophila decrescens* | 126 | 1569 | 879.3492063 | 358.843321 | D | A |
| *Alsophila dregei* | 1288 | 1754 | 1527 | 200.7402966 | D | B |
| *Alsophila dregei* | 1288 | 1754 | 1527 | 200.7402966 | D | B |
| *Alsophila dregei* | 1288 | 1754 | 1527 | 200.7402966 | D | B |
| *Alsophila ferdinandii* | 29 | 128 | 78.44444444 | 36.75973038 | F | A |
| ***Alsophila firma*** | 608 | 2298 | 1279.626943 | 385.0523853 | AB | B |
| ***Alsophila firma*** | 608 | 2298 | 1279.626943 | 385.0523853 | AB | B |
| *Alsophila havilandii* | 2650 | 3199 | 2924.5 | 388.2016229 | E | C |
| *Alsophila imrayana* | 438 | 1858 | 1143.125 | 448.9311566 | ABC | B |
| *Alsophila nigrolineata* | 2166 | 2200 | 2178.666667 | 18.58314649 | F | C |
| *Alsophila oosora* | 1554 | 3199 | 2265.25 | 841.1339073 | E | C |
| *Alsophila ramispina* | 6 | 2686 | 703.7826087 | 671.6294268 | E | A |
| ***Alsophila salvinii*** | 774 | 1982 | 1401.111111 | 334.0709793 | A | B |
| *Alsophila smithii* | 1 | 1396 | 412.8977695 | 289.4965064 | F | A |
| *Alsophila spinulosa* | 464 | 1907 | 1034.8 | 438.4065165 | E | B |
| *Alsophila stelligera* | 50 | 410 | 273.8 | 166.6229276 | E | A |
| ***Alsophila tryoniana*** | 531 | 1978 | 1277.714286 | 403.4910024 | A | B |
| ***Alsophila tryoniana*** | 531 | 1978 | 1277.714286 | 403.4910024 | A | B |
| *Asplenium monanthes* | 21 | 2189 | 1488.701149 | 579.1109391 | ABCD | B |
| *Asplenium praemorsum* | 79 | 2490 | 1505.064516 | 496.9764627 | ABC | B |
| *Azolla mexicana* | 25 | 3021 | 2017.8 | 1189.088601 | A | C |
| *Blechnum glandulosum* | 1006 | 2962 | 1727.409524 | 533.0264767 | AB | B |
| *Blechnum occidentale* | 9 | 1386 | 590.1370787 | 400.3274241 | ABC | A |
| *Blechnum serrulatum* | 1 | 498 | 83.41549296 | 110.590821 | ABC | A |
| *Calochlaena dubia* | 10 | 1200 | 460.1666667 | 356.5289642 | F | A |
| *Calochlaena javanica* | 966 | 1472 | 1207.25 | 173.0234482 | E | B |
| *Calochlaena villosa* | 852 | 2633 | 1941.606061 | 483.2083492 | F | B |
| *Calochlaena villosa* | 852 | 2633 | 1941.606061 | 483.2083492 | F | B |
| *Cibotium barometz* | 344 | 2040 | 1208 | 482.7786242 | E | B |
| *Cibotium glaucum* | 282 | 1962 | 895.8 | 483.2710272 | E | A |
| *Cibotium schiedei* | 42 | 1486 | 1075.916667 | 449.0562747 | A | B |
| *Culcita conniifolia* | 1148 | 3286 | 2384.204819 | 516.6662309 | ABC | C |
| *Culcita conniifolia* | 1148 | 3286 | 2384.204819 | 516.6662309 | ABC | C |
| *Culcita macrocarpa* | 7 | 992 | 324.626087 | 227.53837 | D | A |
| *Cyathea alata* | 25 | 706 | 425.7 | 238.5856706 | F | A |
| ***Cyathea bicrenata*** | 843 | 2153 | 1422.489796 | 320.1516647 | A | B |
| ***Cyathea* sp.** | 843 | 2153 | 1422.489796 | 320.1516647 | A | B |
| *Cyathea caracasana* | 1847 | 2695 | 2168.333333 | 458.6254826 | A | C |
| *Cyathea dejecta* | 191 | 2382 | 1320.444444 | 797.0497336 | B | B |
| ***Cyathea divergens*** | 1042 | 1888 | 1651.297872 | 488.1901609 | AB | B |
| *Cyathea tuerckheimii* | 764 | 2115 | 1434.863636 | 410.2545681 | A | B |
| ***Cyathea divergens*** | 1042 | 1888 | 1651.297872 | 488.1901609 | AB | B |
| ***Cyathea divergens*** | 1042 | 1888 | 1651.297872 | 488.1901609 | AB | B |
| *Cyathea tuerckheimii* | 764 | 2115 | 1434.863636 | 410.2545681 | A | B |
| *Cyathea tuerckheimii* | 764 | 2115 | 1434.863636 | 410.2545681 | A | B |
| *Cyathea farinosa* | 900 | 1956 | 1291.714286 | 405.287846 | B | B |
| *Cyathea farinosa* | 900 | 1956 | 1291.714286 | 405.287846 | B | B |
| *Cyathea fulva* | 637 | 2798 | 1646.60396 | 486.517118 | AB | B |
| *Cyathea fulva* | 637 | 2798 | 1646.60396 | 486.517118 | AB | B |
| ***Cyathea furfuracea*** | 626 | 1703 | 1069.5 | 495.5148837 | C | B |
| *Cyathea godmanii* | 715 | 2046 | 1467.666667 | 300.4350179 | A | B |
| *Cyathea grandifolia* | 87 | 164 | 125.5 | 54.44722215 | B | A |
| *Cyathea grandifolia* | 87 | 164 | 125.5 | 54.44722215 | B | A |
| *Cyathea howeana* | 29 | 90 | 72 | 27.12010324 | F | A |
| *Cyathea hymenophylloides* | 391 | 1616 | 817.3333333 | 444.2182647 | B | A |
| *Cyathea multiflora* | 204 | 1598 | 728.7674419 | 395.2152556 | AB | A |
| ***Cyathea mutica*** | 397 | 2111 | 1188.541667 | 412.0976934 | A | B |
| *Cyathea myosuroides* | 3 | 783 | 232.36 | 226.4931832 | AC | A |
| *Cyathea myosuroides* | 3 | 783 | 232.36 | 226.4931832 | AC | A |
| *Cyathea poeppigii* | 32 | 2173 | 921.1746032 | 573.4471469 | AB | A |
| *Cyathea robertsiana* | 12 | 1190 | 677.0540541 | 317.8508752 | F | A |
| *Cyathea schiedeana* | 419 | 1569 | 902.372549 | 328.9548382 | A | A |
| *Cyathea schiedeana* | 419 | 1569 | 902.372549 | 328.9548382 | A | A |
| *Cyathea senilis* | 317 | 1208 | 760.6666667 | 342.7968742 | B | A |
| *Cyathea stipularis* | 808 | 1950 | 1197.261905 | 301.1007497 | A | B |
| *Dicksonia arborescens* | 760 | 760 | 760 | 0 | C | A |
| *Dicksonia baudouinii* | 659 | 943 | 801 | 200.8183259 | F | A |
| *Dicksonia lanata* | 11 | 1306 | 600.2727273 | 401.235428 | F | A |
| *Dicksonia lanata* | 11 | 1306 | 600.2727273 | 401.235428 | F | A |
| *Dicksonia sellowiana* | 358 | 2469 | 1596.333333 | 579.6841355 | A | B |
| *Dicksonia sellowiana* | 358 | 2469 | 1596.333333 | 579.6841355 | A | B |
| *Diplazium expansum* | 245 | 2515 | 893 | 588.8407821 | ABC | A |
| *Elaphoglossum vestitum* | 527 | 2209 | 1354.444444 | 527.6265514 | AB | B |
| *Lophosoria quadripinnata* | 482 | 3404 | 2037.096916 | 601.3644663 | ABC | C |
| *Lophosoria quadripinnata* | 482 | 3404 | 2037.096916 | 601.3644663 | ABC | C |
| *Loxoma cunninghami* | 6 | 563 | 218.9574468 | 150.9098431 | F | A |
| *Loxoma cunninghami* | 6 | 563 | 218.9574468 | 150.9098431 | F | A |
| *Loxsomopsis pearcei* | 1816 | 3001 | 2430.76 | 335.7386484 | AB | C |
| *Loxsomopsis pearcei* | 1816 | 3001 | 2430.76 | 335.7386484 | AB | C |
| *Marsilea mexicana* | 2 | 4 | 3 | 1.414213562 | A | A |
| *Metaxya rostrata* | 3 | 498 | 181.3703704 | 120.6610298 | AB | A |
| *Metaxya rostrata* | 3 | 498 | 181.3703704 | 120.6610298 | AB | A |
| *Plagiogyria euphlebia* | 7 | 2869 | 731.6781116 | 627.8150075 | E | A |
| *Plagiogyria pectinata* | 453 | 2916 | 1783.285714 | 948.152891 | AB | B |
| *Plagiogyria stenoptera* | 191 | 2072 | 1549.714286 | 308.3735366 | E | B |
| *Plagiogyria yakumonticola* | 834 | 1456 | 1566 | 1172.777778 | E | B |
| *Sphaeropteris albifrons* | 9 | 895 | 395 | 252.2927804 | F | A |
| *Sphaeropteris brunei* | 808 | 2045 | 1279.909091 | 435.6618998 | AB | B |
| *Sphaeropteris capitata* | 1293 | 1819 | 1495.444444 | 173.1344211 | E | B |
| *Sphaeropteris celebica* | 11 | 1980 | 625.4 | 444.0446765 | EF | A |
| *Sphaeropteris excelsa* | 397 | 1612 | 942.1428571 | 547.5543896 | D | A |
| *Sphaeropteris glauca* | 903 | 1612 | 1351 | 249.3471476 | D | B |
| *Sphaeropteris horrida* | 527 | 1834 | 1131.680851 | 397.1670804 | A | B |
| ***Sphaeropteris horrida*** | 527 | 1834 | 1131.680851 | 397.1670804 | A | B |
| *Sphaeropteris medullaris* | 1 | 1080 | 289.7340067 | 174.0461468 | F | A |
| *Sphaeropteris novaecaledoniae* | 402 | 613 | 877 | 503.7272727 | F | A |
| *Sphaeropteris polypoda* | 414 | 1554 | 1819 | 1021.5 | E | B |
| *Sphaeropteris robusta* | 390 | 390 | 390 | 0 | F | A |
| *Thyrsopteris elegans* | 290 | 1000 | 545 | 502.05 | B | A |
| *Thyrsopteris elegans* | 290 | 1000 | 545 | 502.05 | B | A |
| *Woodwardia martinezii* | 1552 | 1901 | 1689 | 193.4657592 | A | B |
| *Woodwardia spinulosa* | 1187 | 2491 | 1888.62069 | 363.5451842 | A | B |
