# Supplementary material for: Historical reconstruction of climatic and elevation preferences and the evolution of cloud forest-adapted tree ferns in Mesoamerica
Source: PeerJ. 2016 Nov 16;4:e2696. doi: 10.7717/peerj.2696 (PMC5119233; doi:10.7717/peerj.2696)
Supplement: Table S3 [file peerj-04-2696-s003.doc]

**Table S3 Factor loadings from the principal components analysis on temperature and precipitation variables from WorldClim.** Factor loadings above 0.5 are indicated in bold.

|  | | | | | | | |
| --- | --- | --- | --- | --- | --- | --- | --- |
|  | **Factor loading** | | | | | | |
| **Variables** | **Tree ferns** | | | **Cyatheaceae** | | | |
|  | **PC1** | | **PC2** |  | **PC1** | **PC2** | |
| Annual mean temperature (BIO1) | | –0.216 | **0.519** |  | –0.130 | **0.658** |  |
| Mean diurnal range (BIO2) | | –0.295 | –0.101 |  | –0.250 | –0.105 |  |
| Isothermality (BIO3) | | 0.022 | **0.885** |  | 0.037 | **0.880** |  |
| Temperature seasonality (BIO4) | | –0.146 | **–0.989** |  | –0.137 | **–0.990** |  |
| Max. temperature of warmest month (BIO5) | | –0.304 | 0.149 |  | –0.206 | 0.373 |  |
| Min. temperature of coldest month (BIO6) | | –0.056 | **0.601** |  | –0.023 | **0.635** |  |
| Temperature annual range (BIO7) | | –0.201 | **–0.890** |  | –0.173 | **–0.879** |  |
| Mean temperature of wettest quarter (BIO8) | | –0.314 | 0.169 |  | –0.183 | 0.353 |  |
| Mean temperature of driest quarter (BIO9) | | –0.192 | **0.697** |  | –0.215 | **0.736** |  |
| Mean temperature of warmest quarter (BIO10) | | –0.293 | 0.197 |  | –0.193 | 0.423 |  |
| Mean temperature of coldest quarter (BIO11) | | –0.136 | **0.731** |  | –0.076 | **0.802** |  |
| Annual precipitation (BIO12) | | **0.998** | –0.054 |  | **0.980** | –0.196 |  |
| Precipitation of wettest month (BIO13) | | **0.922** | 0.133 |  | **0.945** | 0.049 |  |
| Precipitation of driest month (BIO14) | | **0.901** | –0.273 |  | **0.864** | –0.430 |  |
| Precipitation seasonality (BIO15) | | –0.225 | 0.432 |  | –0.182 | **0.576** |  |
| Precipitation of wettest quarter (BIO16) | | **0.932** | 0.134 |  | **0.951** | 0.049 |  |
| Precipitation of driest quarter (BIO17) | | **0.909** | 0.262 |  | **0.869** | –0.425 |  |
| Precipitation of warmest quarter (BIO18) | | **0.890** | –0.136 |  | **0.892** | –0.233 |  |
| Precipitation of coldest quarter (BIO19) | | **0.898** | –0.062 |  | **0.878** | –0.243 |  |
| % of variance explained | | 34.38 | 24.70 |  | 32.40 | 30.82 | |
| ANOVA | | *F*_11, 14544_ = 288.43  *P* < 0.0001 | *F*_11, 14544_ = 1633.82  *P* < 0.0001 |  | *F*_2, 9725_ = 4664.04  *P* < 0.0001 | *F*_2, 9725_ = 52.25  *P* < 0.0001 | |
|  |  | |  |  |  |  | |
