# Supplementary figures and images for: Historical reconstruction of climatic and elevation preferences and the evolution of cloud forest-adapted tree ferns in Mesoamerica
Source: PeerJ. 2016 Nov 16;4:e2696. doi: 10.7717/peerj.2696 (PMC5119233; doi:10.7717/peerj.2696)

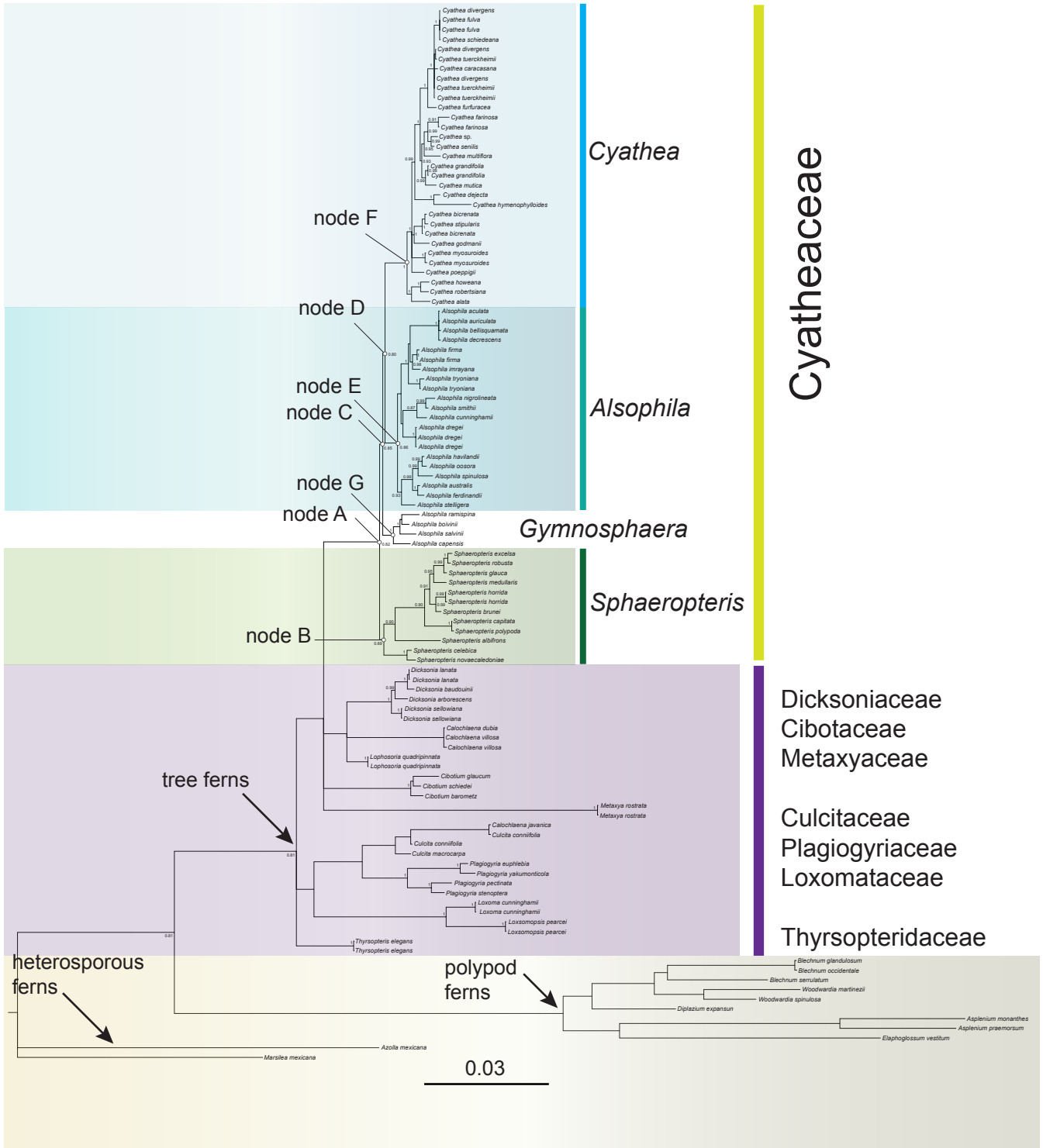

Supplement: Figure S1 [file peerj-04-2696-s004.pdf]

A

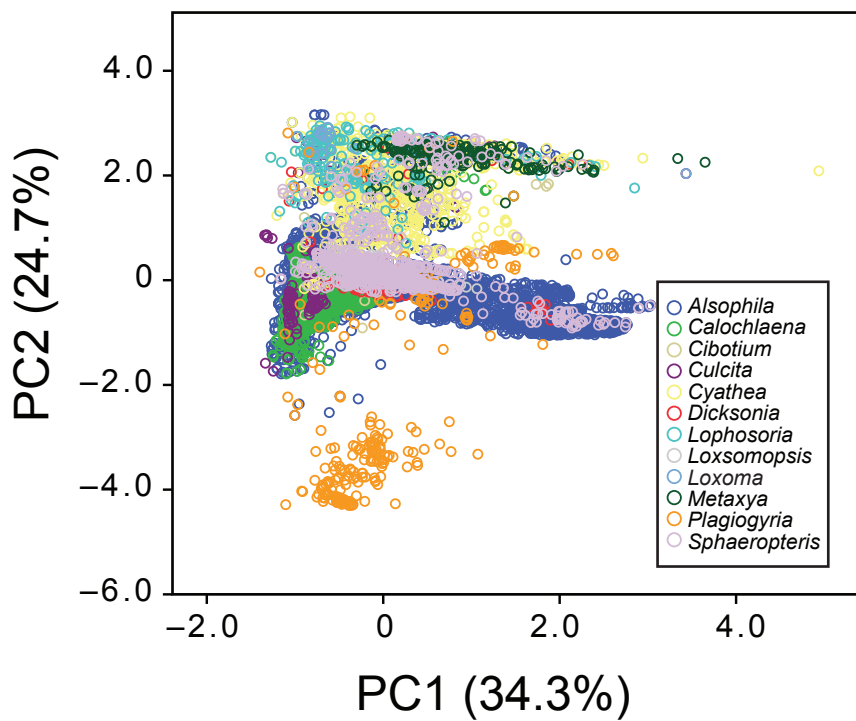

B

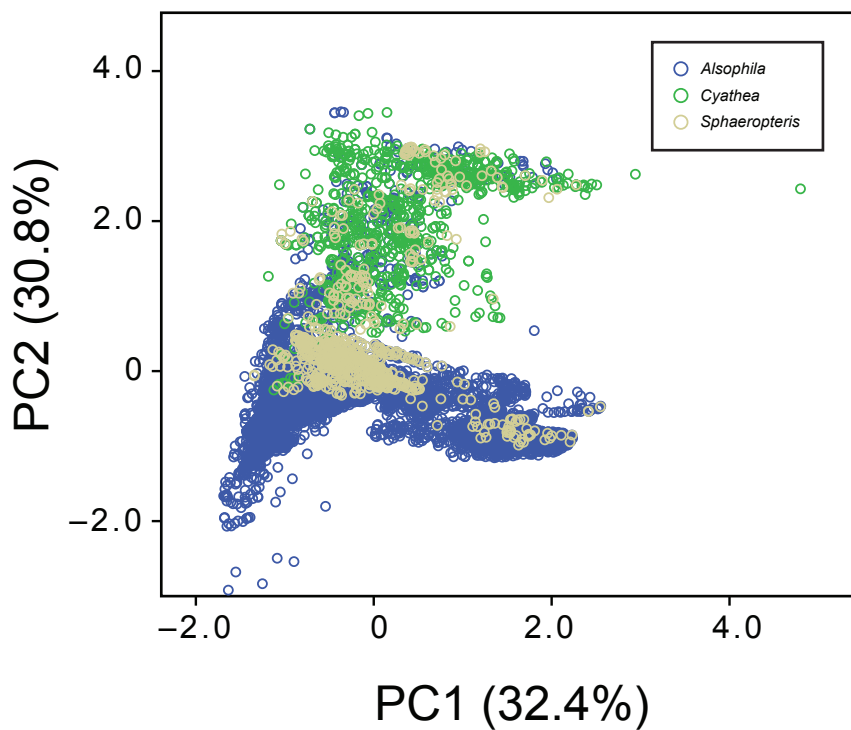

Supplement: Figure S2 [file peerj-04-2696-s005.pdf]
